# Tyrosine Kinase Inhibitors in the Treatment of Systemic Sclerosis: The Difficulty in Interpreting Proof-of-Concept Studies

**DOI:** 10.1155/2011/842181

**Published:** 2011-10-13

**Authors:** Jessica Gordon, Robert Spiera

**Affiliations:** Department of Rheumatology, Hospital for Special Surgery, 535 East 70th Street, New York, NY 10021, USA

## Abstract

Tyrosine kinase inhibitors (TKIs) have emerged as a targeted therapy of interest for the treatment of systemic sclerosis (SSc). Recently, several groups have performed pilot or “proof-of-concept” studies to determine the feasibility of this approach for the treatment of the cutaneous and pulmonary manifestations of this multisystem disease. The conclusions drawn by these different studies have been conflicting, and some controversy has arisen as to whether tyrosine kinase inhibition is a treatment approach worthy of continued study. This paper summarizes this research to date with emphasis on the challenges in interpreting proof-of-concept studies in this patient group.

## 1. Introduction

Systemic sclerosis (SSc) is a heterogeneous, multisystem disorder characterized by vasculopathy, fibrosis, and autoimmunity in the context of both genetic and environmental factors [1]. Although the pathogenesis of SSc is not completely understood, there is strong evidence for a central role of abnormal signaling through the transforming growth factor beta (TGF*β*) pathway [2]. The intracellular tyrosine kinase (TK) c-abl has been implicated in the downstream signaling pathways of TGF*β* [3]. There is additional evidence for abnormalities in the platelet-derived growth factor (PDGF) axis, signaling through the receptor TK the PDGF receptor (R), as contributory to the pathogenesis of SSc as well [4]. 

Because of the roles of these pathways, there has been interest in evaluating the specific tyrosine kinase inhibitors (TKIs) which are able to block c-abl and the PDGFR in the treatment of SSc [5]. These include imatinib mesylate (Gleevec; Novartis Pharmaceuticals; Basel, Switzerland), dasatinib (Sprycel; Bristol Myers Squibb; New York, NY), and nilotinib (Tasigna; Novartis Pharmaceuticals) all of which inhibit c-abl and the PDGFR with different degrees of potency [6]. All three of these medications are FDA approved for the treatment of chronic myelogenous leukemia (CML), and imatinib is additionally FDA approved for the treatment of gastrointestinal stromal tumor (GIST) in addition to other hematologic and oncologic conditions [7–10]. Each medication is additionally capable of inhibiting other kinases. For example, imatinib inhibits c-kit, and dasatinib inhibits src kinases. It is possible that some of these different pathways could prove to be of biologic interest in fibrosing disorders. Each of these TKIs also has slightly different side effect profiles [11], which may also make one or another of them favorable for the treatment of SSc. 

Imatinib, nilotinib, and dasatinib have been shown in *in vitro* models to decrease the TGF-*β* and PDGF-mediated production of extracellular matrix proteins and to abrogate skin thickening observed in mouse models of SSc [12, 13]. Imatinib has also shown antifibrotic properties in preclinical models of renal [14], hepatic [15], and pulmonary fibrosis [16]. Case reports of imatinib have shown promise to this treatment approach [17–21]. To date there have been several “proof-of-concept” (POC) approaches to determine the safety and potential efficacy of TKIs for dcSSc. The inclusion criteria and dosing of the various investigations have varied but the designs are similar as open-label and uncontrolled investigations to assess safety, efficacy, and effect on potential biomarkers of disease. The purpose of this paper is to review these investigations to date with a discussion of the various caveats that come into play with this type of study in SSc. 

It is our opinion that these POC approaches can have great value in the search for improved treatment for this group of patients. However, POC trials pose challenges in study design and have important limitations to the conclusions that can be drawn from them. These POC approaches are appealing because they can utilize a smaller number of patients and provide insight into biologic effect, initial safety, and treatment effect *in vivo*. They can be used as an initial (but not definitive) test for clinical questions and are also an opportunity to test hypotheses with respect to biologic effect of treatment and biomarkers in SSc. The term “proof of concept” or “proof of principle” can hold several meanings. In general the term refers to animal or human studies which can provide evidence for or against a pharmacological or clinical effect. In industry-based drug development these POC trials are used for investment or “go/no go” decisions [22]. 

Multiple aspects of SSc make it difficult to fit into this “go/no go” mold. Firstly, SSc is a rare, heterogeneous, and multisystemic disease. Clinical endpoints and study design vary depending on whether the outcome of interest is cutaneous, pulmonary, vascular, or other. Patient recruitment is difficult because of the rarity of the condition, and the natural history of the involvement of each organ system is variable. Validated clinical endpoints exist for different outcomes, but some, for example, the modified Rodnan Skin Score (mRSS), may be open to bias in an uncontrolled trial, whereas others, for example, the forced vital capacity (FVC), are more objective. Many different biomarkers are under investigation and some have been validated in small populations or under certain circumstances [23]. However, these biomarkers have not yet been tested in clinical trials nor have they been shown to be prognostic at this point in time (with the exception of autoantibodies which have prognostic value at baseline but have not been shown to change with clinical status). The ability to define biomarkers has been further frustrated by the lack of unequivocably efficacious therapies in this condition. POC trials provide an initial insight into whether an agent seems to have acceptable safety and tolerability in the target population and whether there is a suggestion of efficacy that would make a larger double-blind randomized placebo-controlled trial worthwhile as well as how to power such an endeavor. 

 The investigation of TKIs in SSc is a case study of these issues.

### 1.1. Preclinical Studies Assessing Tyrosine Kinase Inhibitors for the Treatment of Fibrosis

Distler et al. showed that the treatment of cultured fibroblasts from both SSc patients and healthy controls with imatinib led to a dose-dependent inhibition of the synthesis of collagen Ia1, collagen Ia2, and fibronectin-1 when the fibroblasts were stimulated with TGF-*β*, PDGF, or left in their baseline state. Using the bleomycin-induced dermal fibrosis mouse model, this group also showed that imatinib treatment both prevented [12] and decreased established cutaneous fibrosis [24]. Using the tight-skin 1 (Tsk1) murine model a similar effect was shown [13]. Similar observations were made with respect to the effects seen in cultured fibroblasts and in the prevention of skin thickening in the bleomycin mouse model with dasatinib and nilotinib [13]. These well-executed studies were essential in establishing the rationale for the clinical study of TKIs in SSc, but it is important to note that both in vitro and animal models of fibrosis frequently fall short in predicting clinical response in SSc. 

Following this work, TKIs have been used in several investigations delineating TGF-*β* signaling and its importance in the fibrotic manifestations of SSc. Pannu et al. observed that activation of TGF-*β*-mediated Smad1 signaling occurs in a subset of SSc patients and contributes to persistent activation of SSc fibroblasts [25]. This Smad1 pathway is inhibited by imatinib. Bhattacharyya et al. delineated a novel signaling mechanism of TGF-*β* involving a transcription factor called early growth response factor 1 (egr1) [26]. Recently, Bujor et al. have shown c-abl is positioned as an upstream regulator of the pro-fibrotic PKC*δ*/P-Fli1 pathway, via induction of PKC*δ* nuclear localization [27]. Li and Jimenez have shown that c-Abl and PKC-*δ* to be important in TGF-*β*-induced endothelial mesenchymal transformation [28]. These pathways are inhibited with imatinib. Additionally, microRNA29 (miR-29) has been observed to be downregulated in SSc fibroblasts, and treatment of these fibroblasts with imatinib reversed the downregulation of miR-29a [29]. Together this body of work presents a very compelling rationale for the investigation of these TKIs for the treatment of fibrosis in SSc and underlines the need for well-designed clinical investigations. These studies postulate specific biologic effects which would be well tested as investigational surrogate markers in a POC study, and collaboration between labs capable of performing such investigations with investigators performing the clinical trials needs to be a focus of the scleroderma research community. 

### 1.2. Treatment of Other Fibrotic Conditions with Imatinib

Imatinib has been investigated for the treatment of various fibrotic diseases apart from systemic sclerosis. These include nephrogenic systemic fibrosis (NSF), sclerodermatous chronic graft-versus-host disease (cGVHD), morphea, and idiopathic pulmonary fibrosis (IPF). Kay and High observed decreased skin thickening and reduction in the mRSS in two patients with NSF [30]. The skin thickening recurred upon cessation of the drug. Olivieri et al. treated 19 patients with refractory cGVHD with fibrotic/sclerotic features and reported an overall response rate at 6 months of 79%, with 7 complete remissions and 8 partial remissions [31]. Similarly, Magro et al. reported a retrospective study of imatinib for sclerodermatous cGVHD where 7 patients responded, 4 of whom had mRSS improvements of 90% or more [32]. Moinzadeh et al. reported a case of one patient with therapy-resistant generalized morphea treated with imatinib with improvement [33]. Imatinib was studied for the treatment of IPF in a double-blind, placebo-controlled, randomized trial [34]. In this study 119 patients were treated with either imatinib or placebo at 600 mg daily (with dose adjustment to 400 mg daily for perceived drug toxicity). A difference was not detected between patients on imatinib compared with placebo with respect to the primary endpoint: time to disease progression as defined as a 10% decline in percent predicted forced vital capacity (FVC) from baseline or time to death. Treatment had no effect on change in forced vital capacity or diffusion capacity of the lungs for carbon monoxide (DLCO) at 48, 72, or 96 weeks. More imatinib-treated patients discontinued study because of perceived drug-related AEs although this did not reach statistical significance (imatinib: 13 versus placebo: 6; *P* = 0.073). These IPF results were discouraging just as the data with sclerodermatous cGVHD and NSF seem encouraging. In both cases, the relation to SSc is uncertain.

### 1.3. Safety and Tolerability from Other Patient Populations

The side effect profiles of tyrosine kinase inhibitors have been described in patients with CML and other malignancies and are summarized in a recent review by Wei et al., and in general this is a relatively well-tolerated medication in this patient group [35]. With respect to imatinib in CML, the most common side effects include myelosuppression, nausea, fatigue, diarrhea, edema, and muscle cramps. Edema may be superficial and responsive to diuretics but also includes periorbital edema, which is less responsive to diuretics. Edema is seen less commonly with dasatinib and nilotinib when compared with imatinib. However, pleural effusion is a more prominent side effect of dasatinib treatment. Nilotinib has been associated with higher rates of dermatologic, hepatic, and pancreatic toxicity when compared with imatinib, but with lower rates of gastrointestinal intolerance, muscle spasm, and neutropenia. 

Cardiotoxicity has been reported in patients with CML treated with imatinib, with some evidence for c-abl inhibition leading to this toxicity [36]. Retrospective, clinical experiences estimate the frequency of this to occur at a rate of 0.5–1.1% with association noted for typical risk factors for cardiovascular disease including age, diabetes, and hypertension [37, 38]. Patients in clinical practice with CHF are able to continue imatinib for malignancies with careful monitoring. In studies of patients with imatinib resistance or intolerance treated with nilotinib, sudden death was reported in 0.6% of patients, and an expanded-access program showed a similar rate of occurrence with concern for possible ventricular repolarization abnormalities because of timing of events [39]. Because of this, the package labeling for nilotinib requires careful EKG monitoring for QTc prolongation with repeat EKGs one week after dose adjustments and guidelines for dose adjustments based on QTc prolongation. 

The side effects described above are important in an SSc population in particular because similar phenomena may be seen also as manifestations of disease activity. Patients with SSc may have occult or overt cardiac involvement with diverse manifestations ranging from conduction abnormality to cardiomyopathy. Not only is it important to carefully monitor for cardiac complications in SSc patients treated with TKIs, but also attribution of these side effects in open-label trials can be difficult. It would require a placebo arm to adequately understand if in SSc certain side effects can also be disease related. Edema, which is seen commonly with imatinib, can be particularly troublesome in a SSc population already uncomfortable with tight and thick skin. This side effect can be confounding in two ways: patients may have edema as an inherent part of early cutaneous manifestations of SSc which and edema can be difficult to distinguish from skin thickness and lead to an elevated measurement of the modified Rodnan Skin Score (mRSS). Myopathy is also common in scleroderma, often with low-grade creatine kinase (CK) elevations. Similar CK elevations have been seen in imatinib use in populations without underlying connective tissue disorders [40]. Attribution of myopathy, myalgias, or even asymptomatic CK elevations in imatinib-treated patients in uncontrolled clinical trials therefore can be similarly problematic. 

Vascular issues are also an issue. PDGFR is on the pericyte and stabilizes blood vessels [41]. Alternatively, PDGF has been implicated in the abnormal proliferation and migration of pulmonary artery smooth muscle cells [42]. This effect in vivo needs to be carefully observed and could be postulated to lead to either efficacy or side effect. Indeed, Hatano et al. treated 5 patients with PAH (3 scleroderma-associated PAH and 2 idiopathic PAH) with imatinib (100 to 200 mg/day) for 24 weeks in addition to standard treatment [43]. However, there was no significant change in hemodynamics or exercise capacity. DLCO improved at 12 and 24 weeks of treatment (*P* < 0.01 and *P* = 0.05), and plasma PDGF-BB levels were significantly decreased after 12 weeks of treatment (*P* = 0.04). Two patients with high plasma PDGF-BB levels had a 15% decrease in pulmonary vascular resistance. Given the multitude of vascular issues in SSc patients, careful observation for vascular effect—positive or negative—needs to be performed carefully. 

### 1.4. Clinical Data Summary—Systemic Sclerosis

There is a solid preclinical rationale for the use of TKIs in SSc; however, the clinical experience in this heterogeneous patient group is still in its early stages. Several case reports, which we have reviewed formerly [44], suggested the clinical benefit for imatinib treatment for the cutaneous and pulmonary manifestations of SSc, and three open-label clinical trials have also been recently reported. None of these were controlled clinical trials, limiting what can be said definitively about either the safety or efficacy of tyrosine kinase inhibition in scleroderma. Other studies have been presented in abstract form at scientific meetings. In addition to the studies that will be discussed below, there is an Italian study still ongoing and a French study completed but not yet reported to our knowledge. There is additionally one trial investigating dasatinib which is completed but not yet reported and one active and recruiting trial at our center investigating nilotinib [45].

 The clinical trials reported to date are heterogeneous in terms of patient populations included, disease duration, imatinib dosing, and study duration. Difficulties in interpreting such open-label proof-of-concept trials with imatinib in scleroderma relate both to generic issues in terms of interpreting outcomes in a disease as heterogenous and variable in its natural history as scleroderma, as well as some issues more uniquely related to imatinib itself, namely the edema that often complicates treatment which can affect the mRSS.

Our group has recently reported an open-label experience treating 30 patients with dcSSc with imatinib for one year [46]. This was a 1-year, single-center, phase 2a, single-arm, open-label clinical trial. Two patient subgroups were prospectively targeted for enrollment: 20 patients with earlier disease (<4 years) and 10 patients with later disease (4–10 years) The mean disease duration based on the time since the first non-Raynaud's symptom attributable to SSc was 3.4 ± 2.3 years for the entire cohort. Mean disease duration in the earlier subgroup was 2.1 ± 1.2 years and in the later subgroup was 6.1 ± 1.6 years. All patients had an mRSS of at least 16, and the mean mRSS at baseline was 30.3 ± 8.7. ILD, which was not a requirement for entry, was present in 53% of patients. Concomitant administration of immunosuppressive agents was not allowed during the course of the trial or in the 3 months prior to enrollment, but 77% of patients had been on various other treatments for dcSSc previously. The target dose of imatinib was 400 mg/daily, but the mean dose tolerated was 300 mg/daily. 24 of 30 patients or 80% tolerated the medication well and completed the trial. Adverse events were common but mostly of grade 1 or 2. Of note, fluid retention which was seen in 80% of patients and GI side effects were similarly common. Neither of these AEs led to discontinuation of imatinib, but often dose adjustment or use of concomitant diuretics was employed. Twenty-four serious AEs were identified, the majority of which were not attributed to imatinib but to SSc itself or to complications from former therapies. 

We observed a decrease in the mRSS by 6.6 points or 22.4% from baseline at 12 months in the 24 completers (*P* = 0.001). After 3 months of treatment there was no change in mRSS, but starting at 6 months of treatment this change started to become evident. (Δ = −4.5; *P* < 0.001 at 6 months). This degree of improvement was seen in patients both early and late-stage diseases. In patients with disease duration of less than 18 months, there was an improvement in the mRSS by 29.5%, in those with disease duration less than 4 years the mRSS declined by 20.1%, and in those with 4 to 10 years of SSc the mRSS improved by 27.8%. There was an improvement of 6.4% predicted (*P* = 0.008) in the FVC, and the diffusion capacity remained stable after 12 months of treatment. The improvement in FVC was significantly greater in patients without interstitial lung disease. In patients with ILD both the FVC and DLCO remained stable over the one-year period of treatment: the mean change in FVC was +2.1% predicted (95% CI −2.9 to 7.0; *P* = 0.36) and the mean change in DLCO was +1.0% predicted (−8.0 to 10.1; *P* = 0.81). High-resolution CTs of the chest, although performed as part of standard of care in most of our patients, were not performed as part of the study protocol and so were not reported. The scleroderma Health Assessment Questionnaire-Disability Index (HAQ-DI) and the short form-36 (SF-36) physical component remained stable over the period of treatment. The SF-36 mental component, the patient global visual analogue scale (VAS), patient shortness of breath VAS, and pain VAS all improved significantly. Dermatopathological assessment demonstrated a significant decrease in skin thickness and improvement in skin morphology in a subset of patients. Histologic improvements paralleled improvements in mRSS. 

Our group's experience is the most extensive published experience of imatinib in the treatment of dcSSc. Definitive conclusions regarding the efficacy of this imatinib for the treatment of dcSSc cannot be made based on this work because of the open-label design. Similarly, attribution of AEs is less certain in this sick patient population in the absence of a parallel placebo-controlled group. A criticism of this trial is that it included both early and later-stage patients, and not only early (<2 years disease duration) diffuse patients as based on the guidelines recommended by White et al. [47]. The rationale for including later-stage patients in this work were that the primary analysis was for safety and that this later-stage group is still very much in need of better treatments for fibrotic manifestations of both the skin and lung. Several facts were encouraging to us: (1) most of our patients were able to complete the trial despite side effects; (2) the improvement in mRSS held true across different disease durations including those with less than 18 months since the time of their first symptom of SSc apart from Raynaud's; (3) histopathology supported our clinical observations; (4) FVC and DLCO were stable in patients with ILD.

Pope et al. presented a 6-month POC trial of imatinib in which they found imatinib to be poorly tolerated at a dose of 200 mg twice daily [48]. Patients with active dcSSc as defined by an increase in mRSS, the presence of tendon friction rubs, or an elevated ESR were recruited. These patients had a mean disease duration of 3.1 years (range 0.3 to 6 years.) The trial was prematurely discontinued after enrollment of 10 patients because of concerns regarding 2 serious adverse effects—fluid retention and weakness, in one patient each. This was designed as a randomized controlled trial with a 4- to 1-randomization scheme and a planned enrollment of 20. Since the trial was terminated early, 9 subjects received active drug and only one received placebo, undermining the study power to compare the efficacy of active treatment versus placebo. Five patients stopped the study medication and needed to interrupt dosage due to adverse events which included fluid retention, weakness, nausea, vomiting, chest pain, worsening anemia, and hair loss. Four patients completed six months of active therapy. The mRSS at baseline was 32.3 and at 6 months was 30.5, which was not statistically different. No significant changes were noted with respect to echocardiogram, PFTs, or other parameters at six months' time. A number of exploratory translational studies from plasma and skin biopsies were reported as not changing significantly from 0 to 6 months other than sVCAM in plasma and sICAM-1 in skin, but data were not provided. An editorial pointed out that these inclusion criteria were somewhat unusual as well as the issue with using this medication in patients with myopathy [49]. This trial allowed concomitant use of methotrexate, which can have pharmacologic interaction with imatinib [50]. In summary, the short duration of the trial and small number of completers make the study inconclusive with respect to efficacy. This group found the tolerability to be poor. 

 Khanna et al. also reported on the use of a higher dose of imatinib (up to 600 mg daily with a mean dose tolerated of 445 mg) in 20 patients with SSc-ILD (14 dcSSc and 6 lcSSc) with treatment duration of 1 year [51]. Twelve of 20 patients discontinued treatment: 7 due to adverse events and 1 was lost to followup. Common AEs (≥20%) included fatigue, edema, nausea, diarrhea, and rash. Proteinuria of a range of 81 to 402 mg/dL was seen in 6 patients without concomitant increase in creatinine. These AEs are described in detail in this study. Adverse effects were felt to be dose related. The authors note that early in the study the first five patients who escalated their dose to 600 mg discontinued the study. The authors later allowed de-escalation or dose stabilization, and in the group that maximally titrated only to 400 mg/daily there were no discontinuations. Treatment with imatinib led to a nonstatistically significant increase in estimated FVC % predicted by 1.74%, TLC % predicted by 4.17%, and DLCO % predicted by 1.46% over 1 year (*P* > 0.05 for all). The mRSS improved by 3.9 units (*P* < 0.001). It is probable that the high level of adverse events noted in this trial relates to the increased dose, and only 30% of patients were able to reach that high of a dose. The improvements in indices of pulmonary function were modest but are worth comparing in magnitude to what was seen in the Scleroderma Lung Study where patients had very similar inclusion criteria where the FVC% predicted decreased by 1% point in the cyclophosphamide-treated arm [52]. Attributing this change to drug effect versus natural history is not possible without a control group, but as an exploratory POC trial this might be considered to justify further investigation in a randomized controlled trial. 

Chung et al. reported in an interim fashion on nine patients treated with lower-dose imatinib at 100 to 200 mg daily [53]. Their cohort included 2 patients with limited SSc with ILD and 7 patients with diffuse SSc. The initial treatment period was 24 weeks. During this time 7 patients were able to complete the course of therapy, 1 patient dropped out secondary to SSc-related keratopathy, and 1 patient with severe ILD died from pneumonia and sepsis. Descriptive reporting of adverse effects included gastrointestinal complaints, edema, and infections. An improvement in mRSS of 32% was recognized in those patients with the diffuse subtype, *P* = 0.005. Evaluation of changes in gene expression by microarray showed a variable cutaneous molecular response to imatinib which might correspond to drug efficacy. This group had previously reported on 2 patients treated with imatinib 200 mg/d with significant improvement in pulmonary and cutaneous parameters [54]. Skin biopsies from these patients before and after treatment demonstrated decreased phosphorylation of PDGF receptor and Abl. Gene expression analysis with microarray on RNA extracted from the skin before and after treatment described the differential expression of 1,050 genes, which led to the hypothesis that there may be an imatinib-responsive gene signature in a specific subgroup of patients with SSc. We look forward to final publication of this experience. This work not only adds to the above experience with imatinib but raises the bigger questions of whether drug therapy influences gene expression in skin and whether we can use gene expression analysis with DNA microarray to predict response to treatment. It is important to emphasize that it will not necessarily completely answer these questions, but instead will provide information which can be considered hypothesis generating. 

Distler et al. reported the results of an industry-sponsored multicenter study which recruited 27 patients to be treated with imatinib initially 200 mg daily to be titrated up to 600 mg daily for six months, then to be observed for an additional 24 weeks of study therapy. This was a more homogenous patient population with respect to disease duration and only patients with dcSSc with less than 18 months since the first non-Raynaud's symptom were enrolled. Only 16 of 27 patients completed 24 weeks of study, and 13 completed their 48-week study visit. The investigators prospectively defined a 25% decrease in mean mRSS at 24 weeks as a positive “proof of concept.” This outcome was not met, and indeed at 24 weeks the mean mRSS increased by 9.9%. At 48 weeks, mRSS was recognized as decreasing by 21%. Levels of collagen Ia1 and fibronectin in skin were reduced with therapy, which might be seen as a positive effect on SSc biomarkers. We hope to see in the future publication of this paper a more extensive treatment of the biological samples obtained. The study utilized a high dose of imatinib, and the fluid retention and edema seen with this medication which is often dose related likely could have confounded interpretation of skin scores leading to an overestimate of skin scores related to edema rather than true skin fibrosis. Although the results at 48 weeks demonstrated decline in skin score, that was a small subgroup of patients, and in those ensuing several months other therapies were permitted making it difficult to know whether the improvement at 48 weeks was related to the earlier imatinib therapy, natural history disease, or effects of other medications. Moreover, with such substantial loss to follow up other patients, it is hard to say whether these results are biased by the greater likelihood of followup in patients who were doing well after exposure to the study intervention. 

Denton et al. performed a retrospective exploratory analysis using these 27 patients from multiple centers as cases compared with well-characterized historical controls from a single-center database matched for age, sex, duration of disease, and baseline mRSS [55]. The change in mRSS observed in Distler et al. was not noted to be different from patients treated with various standard treatments, which in this group meant various active immunomodulatory agents including cyclophosphamide and mycophenolate mofetil treatments in 84% of the controls and no treatment in 16%. How to interpret this comparison is difficult, and there are obvious methodologic difficulties with the use of historical controls. Lumping both active treatments with immunomodulatory regimens with no treatment makes interpretation murky, and it is difficult to draw conclusions. 

The authors of the above studies reach different conclusions regarding both tolerability and potential efficacy of imatinib. Some explanations for these diverse conclusions relate to the different populations studied: patients had different subsets of SSc with different organ manifestations, patients had different durations of disease, different dosages of imatinib were used (in our experience, adverse events, in particular fluid retention, seemed to be dose related, with lower doses of the drug much better tolerated), and different durations of treatment were studied. Moreover, edema can confound the calculation of mRSS which may have been a consideration in the interpretation of the worsening of skin scores at 6 months in Distler et al. in which a high dose (600 mg daily) of imatinib was used. Additionally, it has been recently observed in a pooled analysis of 3 large RCTS that mRSS tends to improve after entering the clinical trials irrespective of the SSc disease duration [56].

## 2. Discussion

There is a strong rationale for the use of TKIs in SSc provided by the preclinical work. The early clinical work shows considerable adverse effects but potential efficacy in at least 3 of 5 the above-discussed trials. Definitive clinical investigation as from a well-designed randomized controlled-trial is yet to be done.

With respect to clinical outcomes, there is great difficulty in the interpretation of these pilot or proof-of-concept trials due to several factors. First and foremost, neither efficacy nor safety can be established with certainty from an uncontrolled experience. With respect to adverse effects, many of the symptoms attributed to medication side effects can also be attributed to the diverse manifestations of SSc, and without a placebo arm, it is not possible to truly interpret this. Secondly, the heterogeneous nature of dcSSc with respect to natural course of the disease further limits these studies' ability to determine efficacy especially if the effect size is small or moderate. If the response to treatment were comparable in magnitude, for example, to the effects of steroids on polymyalgia rheumatica, such conclusions would be clearer. However, even a modest clinical effect would still be welcome in the treatment of systemic sclerosis and is quite difficult to judge in an uncontrolled study. Additionally given the heterogenous nature of the disease, it is possible that a clinical effect would only be seen in a subset of patients, and such a hypothesis is put forth by the work of Chung et al. 

Should open-label POC studies be performed in SSc? It is our opinion that there is value in these pilot studies. They afford initial data on safety in a population which may be very different from the original population tested. With respect to magnitude of response, this early data can be used to develop power calculations for larger, more definitive studies. When coupled to well-designed translational investigations these projects can illustrate important mechanistic concepts. Additionally an open-label trial with all patients receiving active treatment is substantially easier to recruit. Although the POC trials have value, they are inconclusive and specifically challenging in SSc for the reasons discussed above. For exploration of antifibrotic effect, it is unlikely for this to be observed in a short period of time given the half life of collagen, and this type of trial is usually performed over a short period of time. Additionally, there is a paucity of validated surrogate endpoints or biomarkers which can be used in SSc. In the work described above as well as in other early pilot studies in SSc, translational investigations are frequently utilized to explore potential mechanisms of action or to develop new biomarkers for disease activity. This type of work can be considered more hypothesis-generating mechanistic data rather than “proof-of-concept” of a clinical effect at this point in time. 

Ultimately, these Proof-of-Concept open-label trials are inadequate to definitively address efficacy, safety, or even tolerability of TKIs in scleroderma. Those determinations will require the conduct of randomized, double-blinded, placebo-controlled trials. With respect to the use of TKIs in SSc, the available data, although imperfect, suggest that such studies are worth pursuing. 

## References

[1] Varga J, Abraham D (2007). Systemic sclerosis: a prototypic multisystem fibrotic disorder. *Journal of Clinical Investigation*.

[2] Varga JA, Trojanowska M (2008). Fibrosis in systemic sclerosis. *Rheumatic Disease Clinics of North America*.

[3] Rosenbloom J, Castro SV, Jimenez SA (2010). Narrative review: fibrotic diseases: cellular and molecular mechanisms and novel therapies. *Annals of Internal Medicine*.

[4] Gun DD, Distler JHW, Riemekasten G, Distler O (2009). Stimulatory autoantibodies to Platelet-derived growth factor receptors in systemic sclerosis: what functional autoimmunity could learn from receptor biology. *Arthritis and Rheumatism*.

[5] Distler JH, Distler O (2008). Intracellular tyrosine kinases as novel targets for anti-fibrotic therapy in systemic sclerosis. *Rheumatology*.

[6] Okimoto RA, Van Etten RA (2011). Navigating the road toward optimal initial therapy for chronic myeloid leukemia. *Current Opinion in Hematology*.

[7] Cohen MH, Williams G, Johnson JR (2002). Approval summary for imatinib mesylate capsules in the treatment of chronic myelogenous leukemia. *Clinical Cancer Research*.

[8] Bueso-Ramos CE, Cortes J, Talpaz M (2004). Imatinib mesylate therapy reduces bone marrow fibrosis in patients with chronic myelogenous leukemia. *Cancer*.

[9] Kantarjian H, Giles F, Wunderle L (2006). Nilotinib in imatinib-resistant CML and Philadelphia chromosome-positive ALL. *New England Journal of Medicine*.

[10] Talpaz M, Shah NP, Kantarjian H (2006). Dasatinib in imatinib-resistant Philadelphia chromosome-positive leukemias. *New England Journal of Medicine*.

[11] Wei G, Rafiyath S, Liu D (2010). First-line treatment for chronic myeloid leukemia: dasatinib, nilotinib, or imatinib. *Journal of Hematology and Oncology*.

[12] Distler JHW, Jüngel A, Huber LC (2007). Imatinib mesylate reduces production of extracellular matrix and prevents development of experimental dermal fibrosis. *Arthritis and Rheumatism*.

[13] Akhmetshina A, Venalis P, Dees C (2009). Treatment with imatinib prevents fibrosis in different preclinical models of systemic sclerosis and induces regression of established fibrosis. *Arthritis and Rheumatism*.

[14] Wang S, Wilkes MC, Leof EB, Hirschberg R (2005). Imatinib mesylate blocks a non-Smad TGF-*β* pathway and reduces renal fibrogenesis in vivo. *FASEB Journal*.

[15] Yoshiji H, Noguchi R, Kuriyama S (2005). Imatinib mesylate (STI-571) attenuates liver fibrosis development in rats. *American Journal of Physiology&Gastrointestinal and Liver Physiology*.

[16] Daniels CE, Wilkes MC, Edens M (2004). Imatinib mesylate inhibits the profibrogenic activity of TGF-*β* and prevents bleomycin-mediated lung fibrosis. *Journal of Clinical Investigation*.

[17] Sfikakis PP, Gorgoulis VG, Katsiari CG, Evangelou K, Kostopoulos C, Black CM (2008). Imatinib for the treatment of refractory, diffuse systemic sclerosis. *Rheumatology*.

[18] Van Daele PLA, Dik WA, Thio HB (2008). Is imatinib mesylate a promising drug in systemic sclerosis?. *Arthritis and Rheumatism*.

[19] Distler JHW, Manger B, Spriewald BM, Schett G, Distler O (2008). Treatment of pulmonary fibrosis for twenty weeks with imatinib mesylate in a patient with mixed connective tissue disease. *Arthritis and Rheumatism*.

[20] Chung L, Fiorentino DF, BenBarak MJ (2009). Molecular framework for response to imatinib mesylate in systemic sclerosis. *Arthritis and Rheumatism*.

[21] Sabnani I, Zucker MJ, Rosenstein ED (2009). A novel therapeutic approach to the treatment of scleroderma-associated pulmonary complications: safety and efficacy of combination therapy with imatinib and cyclophosphamide. *Rheumatology*.

[22] Lesko LJ, Rowland M, Peck CC, Blaschke TF (2000). Optimizing the science of drug development: opportunities for better candidate selection and accelerated evaluation in humans. *Journal of Clinical Pharmacology*.

[23] Abignano G, Buch M, Emery P, Del Galdo F (2011). Biomarkers in the management of scleroderma: an update. *Current Rheumatology Reports*.

[24] Akhmetshina A, Venalis P, Dees C (2009). Treatment with imatinib prevents fibrosis in different preclinical models of systemic sclerosis and induces regression of established fibrosis. *Arthritis and Rheumatism*.

[25] Pannu J, Asano Y, Nakerakanti S (2008). Smad1 pathway is activated in systemic sclerosis fibroblasts and is targeted by imatinib mesylate. *Arthritis and Rheumatism*.

[26] Bhattacharyya S, Ishida W, Wu M (2009). A non-Smad mechanism of fibroblast activation by transforming growth factor-*β* via c-Abl and Egr-1: selective modulation by imatinib mesylate. *Oncogene*.

[27] Bujor AM, Asano Y, Haines P, Lafyatis R, Trojanowska M (2011). The c-abl tyrosine kinase controls PKC*δ* induced Fli1 phosphorylation in human dermal fibroblasts. *Arthritis & Rheumatism*.

[28] Li Z, Jimenez SA (2011). Protein kinase C *δ* and the c-Abl kinase are required for transforming growth factor-*β* induction of endothelial-mesenchymal transition in vitro. *Arthritis & Rheumatism*.

[29] Maurer B, Stanczyk J, Jüngel A (2010). MicroRNA-29, a key regulator of collagen expression in systemic sclerosis. *Arthritis and Rheumatism*.

[30] Kay J, High WA (2008). Imatinib mesylate treatment of nephrogenic systemic fibrosis. *Arthritis and Rheumatism*.

[31] Olivieri A, Locatelli F, Zecca M (2009). Imatinib for refractory chronic graft-versus-host disease with fibrotic features. *Blood*.

[32] Magro L, Mohty M, Catteau B (2009). Imatinib mesylate as salvage therapy for refractory sclerotic chronic graft-versus-host disease. *Blood*.

[33] Moinzadeh P, Krieg T, Hunzelmann N (2010). Imatinib treatment of generalized localized scleroderma (morphea). *Journal of the American Academy of Dermatology*.

[34] Daniels CE, Lasky JA, Limper AH (2010). Imatinib treatment for idiopathic pulmonary fibrosis: randomized placebo-controlled trial results. *American Journal of Respiratory and Critical Care Medicine*.

[35] Wei G, Rafiyath S, Liu D (2010). First-line treatment for chronic myeloid leukemia: dasatinib, nilotinib, or imatinib. *Journal of Hematology and Oncology*.

[36] Kerkelä R, Grazette L, Yacobi R (2006). Cardiotoxicity of the cancer therapeutic agent imatinib mesylate. *Nature Medicine*.

[37] Atallah E, Durand JB, Kantarjian H, Cortes J (2007). Congestive heart failure is a rare event in patients receiving imatinib therapy. *Blood*.

[38] Breccia M, Cannella L, Frustaci A, Stefanizzi C, Levi A, Alimena G (2008). Cardiac events in imatinib mesylate-treated chronic myeloid leukemia patients: a single institution experience. *Leukemia Research*.

[39] Novartis Tasigna (nilotinib) US prescribing information.

[40] Gordon JK, Magid SK, Maki RG, Fleisher M, Berman E (2010). Elevations of creatine kinase in patients treated with imatinib mesylate (Gleevec). *Leukemia Research*.

[41] Winkler EA, Bell RD, Zlokovic BV (2010). Pericyte-specific expression of PDGF beta receptor in mouse models with normal and deficient PDGF beta receptor signaling. *Molecular Neurodegeneration*.

[42] Barst RJ (2005). PDGF signaling in pulmonary artery hypertension. *The Journal of Clinical Investigation*.

[43] Hatano M, Yao A, Shiga T, Kinugawa K, Hirata Y, Nagai R (2010). Imatinib mesylate has the potential to exert its efficacy by down-regulating the plasma concentration of platelet-derived growth factor in patients with pulmonary arterial hypertension. *International Heart Journal*.

[44] Gordon J, Spiera R (2011). Imatinib and the treatment of fibrosis: recent trials and tribulations. *Current Rheumatology Reports*.

[45] http://www.clinicaltrials.gov/ct2/show/study/NCT00764309?term=dasatinib+in+scleroderma&rank=1.

[46] Spiera RF, Gordon JK, Mersten JN (2011). Imatinib mesylate (Gleevec) in the treatment of diffuse cutaneous systemic sclerosis: results of a 1-year, phase IIa, single-arm, open-label clinical trial. *Annals of Rheumatic Diseases*.

[47] White B, Bauer EA, Goldsmith LA (1995). Guidelines for clinical trials in systemic sclerosis (scleroderma). I. Disease-modifying interventions. The American College of Rheumatology Committee on Design and Outcomes in Clinical Trials in Systemic Sclerosis. *Arthritis & Rheumatism*.

[48] Pope J, McBain D, Petrlich L Imatinib in active diffuse systemic sclerosis: a single site trial.

[49] Mendoza FA, Jimenez SA Tyrosine kinase inhibitor therapy for systemic sclerosis: quo vadis?.

[50] Van Hest RM, Schnog JB, Van’t Veer MB, Cornelissen JJ (2008). Extremely slow methotrexate elimination in a patient with t(9;22) positive acute lymphoblastic leukemia treated with imatinib. *American Journal of Hematology*.

[51] Khanna D, Saggar R, Mayes MD Open-label pilot trial of imatinib mesylate (Gleevec) in the treatment of systemic sclerosis- associated active interstitial lung disease (SSc-ILD).

[52] Tashkin DP, Elashoff R, Clements PJ (2006). Cyclophosphamide versus placebo in scleroderma lung disease. *The New England Journal of Medicine*.

[53] Chung L, Ruiz P, Wood T Evaluation of an imatinib response gene signature in patients with systemic sclerosis [abstract].

[54] Chung L, Fiorentino DF, BenBarak MJ (2009). Molecular framework for response to imatinib mesylate in systemic sclerosis. *Arthritis and Rheumatism*.

[55] Denton CP, Nihtyanova SI, Varga J Comparative analysis of change in modified Rodnan skin score in patients with diffuse systemic sclerosis receiving imatinib mesylate suggests similar disease course to matched patients receiving standard therapy [abstract].

[56] Amjadi S, Maranian P, Furst DE (2009). Investigators of the D-Penicillamine, Human Recombinant Relaxin, and Oral Bovine Type I Collagen Clinical TrialsCourse of the modified Rodnan skin thickness score in systemic sclerosis clinical trials: analysis of three large multicenter, double-blind, randomized controlled trials. *Arthritis & Rheumatism*.

